# Allelotype of 28 human breast cancer cell lines and xenografts

**DOI:** 10.1038/sj.bjc.6601448

**Published:** 2003-12-09

**Authors:** I C Harkes, F Elstrodt, W N M Dinjens, M Molier, J G M Klijn, E M J J Berns, M Schutte

**Affiliations:** 1 Department of Medical Oncology, Josephine Nefkens Institute, Erasmus University Medical Center, PO Box 1738, 3000 DR Rotterdam, The Netherlands; 2 Department of Pathology, Josephine Nefkens Institute, Erasmus University Medical Center, PO Box 1738, 3000 DR Rotterdam, The Netherlands

**Keywords:** allelic loss, breast cancer, cell lines, loss of heterozygosity, xenografts

## Abstract

Heterozygous loss of relatively large chromosomal regions is a hallmark of the inactivation of tumour suppressor genes. Searching for deletions in cancer genomes therefore provides an attractive option to identify new tumour suppressor genes. Here, we have performed a genome-wide survey for regions exhibiting allelic loss in 24 commercially available breast cancer cell lines and four breast cancer xenografts, using microsatellite analysis. The assembled allelotype revealed an average fractional allelic loss of 0.34. A total of 19 arms had low allelic loss frequencies (<25%) and 17 arms had moderate allelic loss frequencies (25–50%). Five chromosomal arms were deleted in more than half of the breast cancer samples (8p, 10q, 13q, 17p, and 17q). Three of these frequently lost chromosomal arms had not been identified as such by comparative genome hybridisation, illustrating the higher sensitivity of microsatellite analysis for the detection of allelic losses. As we present allelic loss data of individual samples, our allelotype should not only aid the identification of new breast cancer genes but also provides a baseline for myriad studies involving these breast cancer cell lines.

Genetic analyses of sporadic breast cancers have thus far identified few archetypal tumour suppressor genes involved in the development of these tumours. The *p53* tumour suppressor gene was found to be mutated in 20–40% of breast cancers, and the *RB1* and *E-cadherin* genes were inactivated in 10–20% of tumours (Lee *et al*, 1988; T'Ang *et al*, 1988; Hollstein *et al*, 1991; Berns *et al*, 1995, 2000; Berx *et al*, 1995, 1996; van de Wetering *et al*, 2001). Other tumour suppressor genes were mutated in only a minority of sporadic breast cancers (<10%); that is, *APC*, *ATM*, *BRCA1, BRCA2, MAP2K4*, *PTEN*, *p16*, *SMAD4*, *VHL*, and the mismatch repair genes *MLH1*, *MSH2*, and *MSH6*.

The biallelic inactivation of tumour suppressor genes generally involves a small intragenic alteration in one copy of the gene, combined with the loss of a larger chromosomal region containing the other allele. Although the smaller mutations may present problems in detection, the larger regions of deletion are readily identifiable by genomic screening. An allelotype is a genome-wide survey for regions that are characteristically deleted in a tumour type (Vogelstein *et al*, 1989). Such identified regions of frequent allelic loss are thought to be indicative of the location of tumour suppressor genes. Here, we present an allelotype of 24 commercially available breast cancer cell lines and four breast cancer xenografts.

## MATERIALS AND METHODS

### Samples

**Figure 1:**
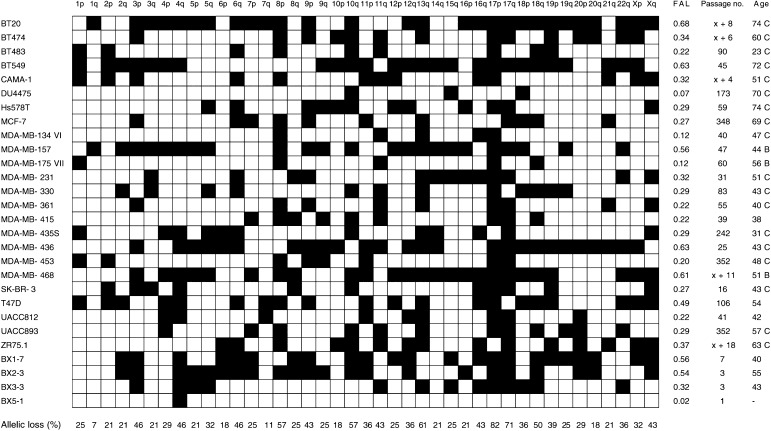
Presumptive allelic losses of human breast cancer samples. In all 24 ATCC breast cancer cell lines and four xenografts (with BX-identifiers) are listed on the left and 41 non-acrocentric chromosomal arms are listed on the top. White squares: both alleles had been retained; black squares: allelic loss was presumed; FAL: fractional allelic loss; passage no.: number of passages that the tumour cells had been propagated *in vitro* or *in vivo*, where x indicates an unknown number of passages that the cells had undergone prior to culture in our laboratory; age: age of the patients at breast cancer diagnosis or resection of the tumour cells, followed by their ethnicity where "C" and "B" indicate Caucasian and Black, respectively.

The 28 human breast cancer samples used in this study are listed in Figure 1

. All 24 breast cancer cell lines were obtained from American Type Culture Condition and were grown according to their recommendations. The four breast cancer xenografts were generated by implantation of breast cancer specimens into immune-deficient mice, as described (Sakakibara *et al*, 1996). Family history of breast cancer or *BRCA1* and *BRCA2* mutational status are not known for any of the breast cancer samples. Controls (*n*=25) were non-neoplastic blood or tissue samples from randomly selected, apparently healthy Dutch individuals. Genomic DNA was extracted using the Qiagen DNeasy kit. Microsatellite analyses indicated that all samples were unique and monoclonal. Of the 28 breast cancer samples, 22 (79%) were shown to be heterozygous for X-chromosome microsatellites (Figure 1). Y-chromosome microsatellite *DYS200* was detected in all male-derived controls, but not in any of the breast cancer samples nor in the female-derived controls.

### Microsatellite analysis

Microsatellites were PCR-amplified as previously described (van de Wetering *et al*, 2001). PCR products were separated by electrophoresis in standard denaturing sequencing gels and the reactions were visualised by autoradiography. All reactions were scored by at least two investigators who were unaware of each other's scores. When the scores were discordant, data were re-evaluated and reactions were repeated when desired. Primer sequences are available upon request.

## RESULTS AND DISCUSSION

We analysed 24 commercially available breast cancer cell lines and four breast cancer xenografts for allelic losses at all 41 non-acrocentric chromosomal arms, by PCR amplification of microsatellites. A single locus was analysed for each chromosomal arm, encompassing a known tumour suppressor gene at 5q21 (*APC*), 9p21 (*p16*), 10q23 (*PTEN*), 13q12 (*BRCA2*), 16p13 (*AXIN1*), 16q22 (*E-cadherin*), 17p13 (*p53*), 17q21 (*BRCA1*), 18q21 (*SMAD4*), 19p13 (*STK11*), and 22q11 (*hSNF5*). Since constitutional non-neoplastic tissues were not available for any of the breast cancer samples, allelic losses were presumed based on statistical arguments. Multiple microsatellite markers were therefore designed within a 5-centiMorgan locus, thus reducing the odds of a deletion breakpoint between markers within a locus. Analysis of genomic DNA from 25 randomly selected non-neoplastic control samples revealed heterozygosity ratios for all microsatellite markers. Heterozygosity ratios generally were about 0.1 lower than those reported in the NCBI database, but higher ratios were also observed. The number of microsatellites that was analysed for each locus was such that the probability for a heterozygous sample to have a single allele size for each of the microsatellites within the locus was less than 5%, based on the heterozygosity ratios of the used markers. Allelic loss of a chromosomal locus was presumed when a breast cancer sample had a homozygous allele pattern for all microsatellites within the locus (with *P*<0.05 for each locus). In total, 146 microsatellites were analysed, resulting in an average of 3.6 markers per locus and an average *P*-value of 0.01. A homozygous allele pattern was observed for the heterozygous control samples at eight of 986 (1%) analysed loci, thus validating the statistical approach and implying an overall error rate for the complete allelotype of about 1%.

The presumptive allelic losses of all 28 breast cancer samples are detailed in Figure 1. The fractional allelic loss (FAL; i.e., the fraction of chromosomal arms that are lost in a particular sample (Vogelstein *et al*, 1989)) ranged from 0.02 for xenograft BX5-1 to 0.68 for cell line BT20 (one and 28 of a total of 41 arms, respectively). The average FAL among all 28 breast cancer samples was 0.34, with an apparently normal distribution pattern of FAL values. This distribution pattern is in contrast to that seen for colorectal cancer, for example, where tumours with the microsatellite instability (MIN) phenotype had distinctly lower FAL values than tumours with the chromosome instability (CIN) phenotype (Lengauer *et al*, 1998). Analysis of the 28 breast cancer samples from our cohort with markers diagnostic for the MIN phenotype (BAT25, BAT26, and BAT40) revealed two breast cancer samples each with an instable length of only the BAT40 marker (i.e., MDA-MB-157 and MDA-MB-175VII), whereas the remaining samples all had stable lengths of all three BAT markers. The exclusive CIN phenotype among the 28 breast cancer samples from our cohort is likely reflected in the relatively high average FAL of 0.34 among these samples (Lengauer *et al*, 1998; Orr-Weaver and Weinberg, 1998; Loeb, 2001).

**Figure 2:**
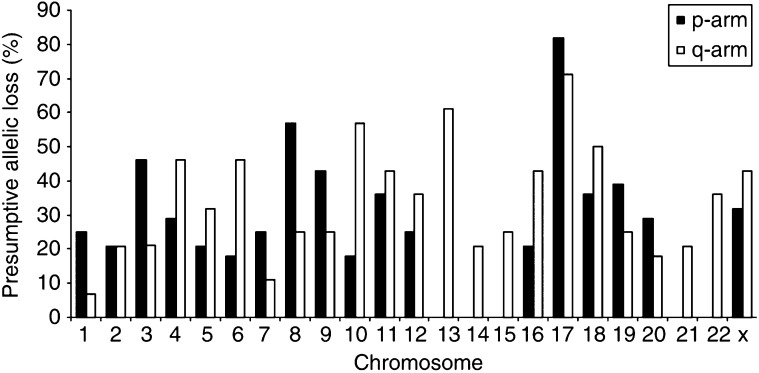
Allelotype of 28 human breast cancer cell lines and xenografts.

A compilation of the presumptive allelic losses of the 28 breast cancer samples is shown in Figure 2

. Low allelic loss frequencies (⩽25% of the samples) were seen at 19 chromosomal arms and 17 chromosomal arms had moderately frequent allelic losses (25–50% of the samples). Five chromosomal arms were lost in more than half of the samples (8p, 10q, 13q, 17p, and 17q). It should be noted that our allelic loss frequencies are conservative estimates of the true extent of allelic losses, as all subchromosomal deletions not involving the analysed locus would go undetected.

**Figure 3:**
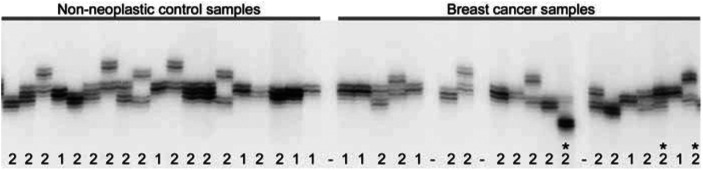
Microsatellite analysis of marker *D8S88* in breast cancer samples and non-neoplastic control samples. 1: one allele size detected; 2: two allele sizes detected; asterix: possible allelic imbalance due to genomic amplification of the locus.

Several breast cancer allelotypes have been reported (Kerangueven *et al*, 1997; Mertens *et al*, 1997; Davidson *et al*, 2000; Forozan *et al*, 2000; Kytola *et al*, 2000; Osborne and Hamshere, 2000). In general, frequencies of allelic loss reported in the literature are lower than the frequencies identified in our cohort of breast cancer samples. Discrepancies in allelic loss frequencies may have three, not mutually exclusive, reasons. First, allelic losses could result from genetic instability due to selective pressures during *ex vivo* propagation of the breast cancer cells, either in cell culture or as xenograft. Deletions of genetic material could occur at any time during propagation of the tumour cells or be most pronounced early in establishing the cell lines or xenografts. In our cohort of breast cancer cell lines, FAL values did not correlate with the number of culture passages (Figure 1), arguing against artefactual allelic deletions during *in vitro* propagation. The five cell lines with extreme high passage numbers had, in fact, FAL values below the average FAL of 0.34 (i.e., DU4475, MCF-7, MDA-MB-435S, MDA-MB-453, and UACC893; Figure 1). As original (preculture or prexenograft) tumour specimens were not available for any of the 28 breast cancer samples from our cohort, we can not, however, conclusively exclude artefactual allelic deletions. A single report has appropriately addressed the issue of artefactual *in vitro* deletions (Wistuba *et al*, 1998). Cell lines of 17 breast cancer specimens had been established and the cells had been propagated *in vitro* for a median period of 2 years. Comparison of 867 microsatellite analyses of the 17 cell lines to those of their corresponding archival tumour specimens revealed an overall concordance of 96%, indicating that allelic deletion at any time during *in vitro* propagation of the breast cancer cells is negligible. As for xenografts, studies on pancreatic cancer have shown that allelic deletions do not occur during *in vivo* propagation of tumour cells in immune-deficient mice (Caldas *et al*, 1994; Hahn *et al*, 1995). Whereas the occurrence of artefactual allelic deletions during *ex vivo* propagation of cancer cells has often been suggested, evidence for such genetic instability has, to the best of our knowledge, never been provided. A second, more likely reason for the higher frequencies of allelic loss in our allelotype is a more accurate microsatellite analysis of these breast cancer samples, as they all consist solely of neoplastic human cells. An extensive host desmoplastic response to the tumour cells is indeed characteristic of most breast cancers, resulting in an admixture of non-neoplastic cells in the tumour specimens that will severely hamper genetic analyses. Imbalances in allele quantities in microsatellite analyses (illustrated in Figure 3

) always reflect genomic amplifications when pure tumour samples are used, such as our breast cancer cell lines or xenografts. We observed allelic imbalances for multiple microsatellite markers only at chromosomal arm 8q for six of the 28 breast cancer samples and at 20q for two samples, whereas imbalances at other loci were only occasionally observed. When primary breast cancer specimens are used, however, allelic imbalances may also be due to residual non-neoplastic cells in the samples, resulting in a major amplification product of the retained allele and a minor amplification product of the lost allele. The non-neoplastic cell content in tumour specimens may even be as such that allelic losses go undetected. Allelic loss frequencies in primary breast cancer specimens will therefore generally be lower than those obtained for cell lines, xenografts, or micro-dissected tumours. As for the five chromosomal arms that we found to be most frequently deleted, comparable frequencies of allelic loss (>50%) in breast cancer specimens have been reported for 8p, 17p, and 17q, indicating that our allelotype indeed reflects genomic deletions as they occur during breast tumorigenesis (Devilee *et al*, 1989, 1991a, 1991b; Sato *et al*, 1990, 1991; Cornelis *et al*, 1994; Aldaz *et al*, 1995; Bieche and Lidereau, 1995; Fujii *et al*, 1996; Kerangueven *et al*, 1997; Anbazhagan *et al*, 1998; Fukino *et al*, 1999; Forozan *et al*, 2000; Shen *et al*, 2000). Third, allelic losses may be masked in karyotype-based techniques, due to genomic amplifications or chromosomes that are aberrantly duplicated through nondisjunction at cellular division. Indeed, two or more copies of a chromosome have been observed in tumour karyotypes, even though molecular analysis with polymorphic markers had shown that those tumours had loss of heterozygosity for that particular chromosome (Lengauer *et al*, 1998). As the comparative genome hybridisation (CGH) technique does not distinguish between paternal and maternal chromosomes, this method is more likely to underestimate allelic loss frequencies than microsatellite analysis. Comparison of our allelotype with three studies that also included the ATCC breast cancer cell lines revealed that the CGH studies had not detected one-quarter to half of the chromosomal arms with allelic loss that we identified using microsatellite analysis (Davidson *et al*, 2000; Forozan *et al*, 2000; Kytola *et al*, 2000). Comparison of the studies is, however, somewhat problematic. Our allelotype, for example, had not detected about one-quarter of the losses identified by CGH, presumably related to the limited resolution of our allelotype (one locus per chromosomal arm). There were also differences among the three CGH studies. Most striking was the consistent underestimation of the allelic losses at 17p and 17q by CGH, likely due to several regions of amplification that are known to be present at this chromosome, such as the *ERBB2* gene at 17q12. Losses at 10q and 13q were variably underestimated, while loss at 8p was among the most frequent losses in all studies.

The wide range from 7% allelic loss at 1q to 82% at 17p that we observed in our breast cancer allelotype indicated a nonrandom deletion of genetic material, suggestive of the location of tumour suppressor genes at the sites of frequent allelic loss. Chromosomal arm 17p contains the *p53* and *MAP2K4* tumour suppressor genes that were mutated in 20 (71%) and three (11%) of the 28 breast cancer samples, respectively, with one tumour being double mutant (Wasielewski and Schutte, *manuscript in preparation*, and (Su *et al*, 2002). Together these mutation account for 88% of the 17p deletions positional candidate genes for the frequent allelic losses at 10q, 13q, and 17q are *PTEN*, *BRCA2* and *RB1*, and *BRCA1*. Systematic mutational analyses of these tumour suppressor genes in this cohort of commercially available breast cancer samples should reveal whether these known genes indeed were the targets of the frequent allelic losses, or that other new tumour suppressor genes are likely to be located at these chromosomal sites.
